# Evaluation of Magnetic Nanoparticle-Labeled Chondrocytes Cultivated on a Type II Collagen–Chitosan/Poly(Lactic-co-Glycolic) Acid Biphasic Scaffold

**DOI:** 10.3390/ijms18010087

**Published:** 2017-01-04

**Authors:** Juin-Yih Su, Shi-Hui Chen, Yu-Pin Chen, Wei-Chuan Chen

**Affiliations:** 1 Department of Surgery, Chang Gung Memorial Hospital, Keelung 204, Taiwan; su1491@adm.cgmh.org.tw (J.-Y.S.); zoe0xo@gmail.com (S.-H.C.); 2 Department of Orthopaedic Surgery, Wan Fang Hospital, School of Medicine, College of Medicine, Taipei Medial University, Taipei 116, Taiwan; yupinjames@yahoo.com.tw; 3 Graduate School of Biotechnology and Bioengineering, Yuan Ze University, Chung-Li, Taoyuan 320, Taiwan

**Keywords:** chondrocytes, type II collagen, chitosan, poly(lactic-co-glycolic acid), magnetic nanoparticles, biphasic scaffold

## Abstract

Chondral or osteochondral defects are still controversial problems in orthopedics. Here, chondrocytes labeled with magnetic nanoparticles were cultivated on a biphasic, type II collagen–chitosan/poly(lactic-co-glycolic acid) scaffold in an attempt to develop cultures with trackable cells exhibiting growth, differentiation, and regeneration. Rabbit chondrocytes were labeled with magnetic nanoparticles and characterized by scanning electron microscopy (SEM), transmission electron (TEM) microscopy, and gene and protein expression analyses. The experimental results showed that the magnetic nanoparticles did not affect the phenotype of chondrocytes after cell labeling, nor were protein and gene expression affected. The biphasic type II collagen–chitosan/poly(lactic-co-glycolic) acid scaffold was characterized by SEM, and labeled chondrocytes showed a homogeneous distribution throughout the scaffold after cultivation onto the polymer. Cellular phenotype remained unaltered but with increased gene expression of type II collagen and aggrecan, as indicated by cell staining, indicating chondrogenesis. Decreased SRY-related high mobility group-box gene (*Sox-9*) levels of cultured chondrocytes indicated that differentiation was associated with osteogenesis. These results are encouraging for the development of techniques for trackable cartilage regeneration and osteochondral defect repair which may be applied in vivo and, eventually, in clinical trials.

## 1. Introduction

Articular cartilage defects related to congenital deficiency, trauma, or sports injury are common problems. However, to date, repair of articular defects remains a significant challenge, since cartilage damage has a poor intrinsic capacity for healing [1]. For orthopedic surgeons, the goal of treatment is to achieve regeneration of organized hyaline cartilage instead of fibrocartilage [2,3,4]. Various techniques exist for chondral or osteochondral defect treatment, including abrasion, drilling, microfracture, osteochondral autografts, osteochondral allografts, and chondrocyte transplantation, but the effectiveness of these varies [3,4]. Osteochondral autologous transplantation is the current treatment of choice for articular cartilage defect repair with proven long-term effectiveness [2,5]. However, limited graft availability and donor site morbidity are still concerns.

A potentially attractive alternative is to use a biomaterial scaffold that simplifies the surgical procedure and which are readily available without causing donor site morbidity [6]. Chondrocytes or differentiated mesenchymal stem cells can then be seeded as a bio-scaffold in cartilage tissue engineering [7,8]. Regeneration with cell-based, tissue-engineering constructs has proven effective in the treatment of cartilage and meniscal defects in several studies [7,9,10]. However, heterogeneous cell distributions throughout the three-dimensional (3D) scaffold under different conditions of cell seeding and culturing require further investigation [11].

Recently, magnetic nanoparticles have been utilized as cell markers and for tracking to investigate mechanisms of tissue regeneration [12,13]. Magnetic nanoparticles have been proven to be non-toxic and have no deleterious effects on cartilage viability [14]. Moreover, Shimizu et al. [15] demonstrated a methodology for cell seeding into 3-D porous hydroxyapatite scaffolds using magnetism to propel the magnetic nanoparticles tags. Labeled bone marrow stromal cells were successfully distributed homogeneously throughout the internal space of scaffolds with high cell densities. However, there is still a lack of studies on the effectiveness of magnetically labeled chondrocytes incorporation into 3D scaffolds under the influence of magnetic fields.

Considering magnetic nanoparticles as cell labels and guided-seeding for cartilage tissue engineering, a bi-layer scaffold composed of chitosan and type II collagen fused with poly(lactic-co-glycolic) acid (PLGA) was developed in this study. We hypothesized that the chondrocytes labeled with magnetite nanoparticles would be homogeneously incorporated into the bi-layer scaffold by the magnetic field. The aims of this study were to improve the efficiency of seeding cells onto the scaffold construct and to allow for cell tracking in articular cartilage engineering.

## 2. Results

### 2.1. Chondrocyte Labeling with Magnetic Nanoparticles

**Figure 1:**
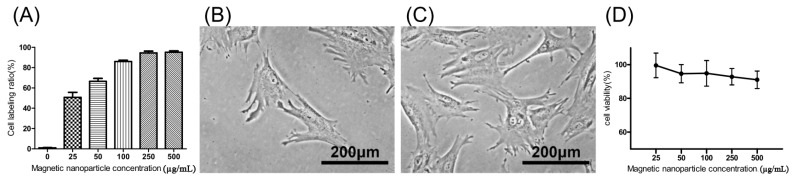
(**A**) Chondrocyte labeling between 0 and 500 µg/mL of magnetic nanoparticles in culture medium in a time-dependent manner three times; (**B**) Cell morphology of unlabeled chondrocytes (0 µg/mL); (**C**) 250 µg/mL; and (**D**) cell viability of labeled chondrocytes in a time-dependent manner three times. Results are presented as % of 0 µg/mL of magnetic nanoparticles. Each value is shown using the mean ± standard errors of the mean (SEM) of three determinations.

**Figure 2:**
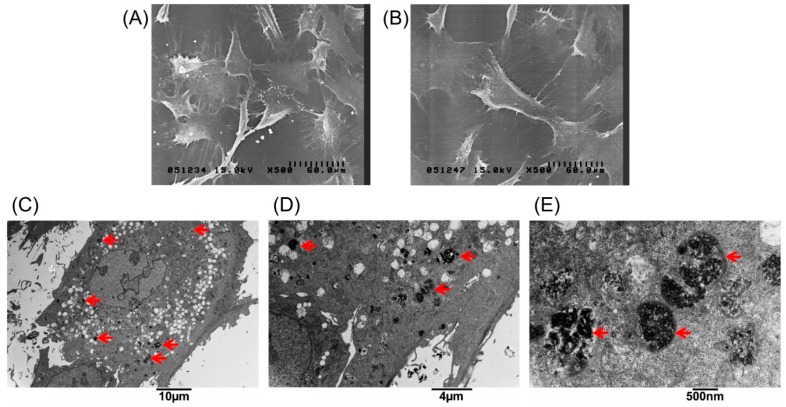
Scanning electron microscopic images of (**A**) unlabeled chondrocytes; and (**B**) labeled with 250 µg/mL magnetic nanoparticles. Transmission electron microscopy (TEM) analysis to demonstrate cellular uptake and subcellular localization of magnetic nanoparticles in chondrocytes. (**C**–**E**) Chondrocytes treated with 250 µg/mL magnetic nanoparticles for 24 h, fixed and stained for TEM. The images were taken at different magnifications. The arrows indicate the locations of magnetic particles in the cells.

**Figure 3:**
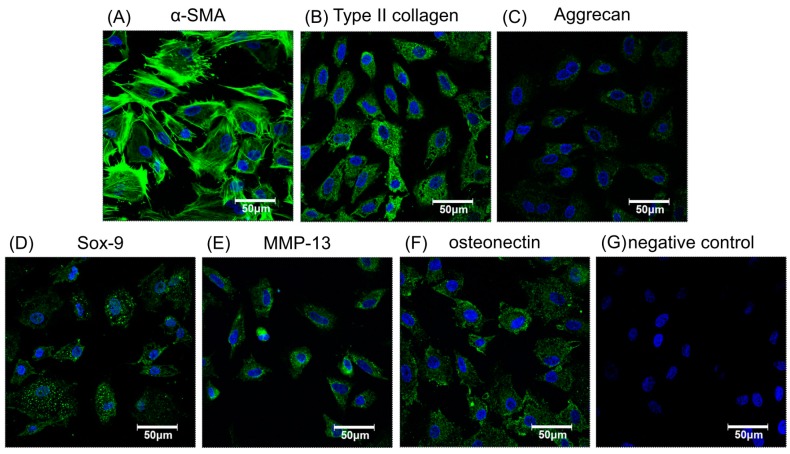
The immunofluorescence staining of chondrogenic- or osteogenic-related proteins to verify phenotypic functions of chondrocytes labeled by 250 µg/mL magnetic nanoparticles. Chondrocytes stained for nuclei (blue) and α-SMA (green) to show cell morphology (**A**). Cells stained with chondrogenic related antibodies to collagen type II (**B**), aggrecan (**C**), and Sox-9 (**D**). Stained with osteogenic related antibodiesMMP-13 (**E**), osteonectin (**F**), and the negative control (**G**). Green indicates specific antibodies, blue, the nucleus.

The extent of labeled and unlabeled chondrocytes was evaluated together with any changes in morphology and chondrogenic potential using electron microscopy, immunofluorescence staining, and genes expressed. The experimental results in Figure 1A, showed that the chondrocytes were labeled successfully by incorporation of magnetic nanoparticles in a time-dependent manner three times. The result showed that the incorporation ratio of magnetic nanoparticles reached a plateau (approximately 95%, 43,454 ± 6270 cells) when the concentration of magnetic nanoparticles exceeded 250 µg/mL. The labeling by iron oxide nanoparticles was found not to affect chondrocytes phenotype or morphology. Figure 1B,C clearly demonstrate similar cellular morphologies of both unlabeled and labeled chondrocytes. The cell toxicity of the iron oxide nanoparticles was also evaluated. Figure 1D shows that the cell viability following labeling using between 25 and 500 µg/mL of iron oxide nanoparticles were from 100% to 90% in a time-dependent manner three times. This experiment proves that the iron oxide nanoparticles do not affect cell viability or proliferation. Therefore, the concentration of 250 µg/mL of magnetic nanoparticles was used in the following experiments. Figure 2A,B, scanning electron microscopic (SEM) images, indicated the same unchanged cellular morphology after labeling with magnetic nanoparticles. Magnetic labeling using 250 µg/mL had no adverse effects on cell viability when compared to non-labeled controls (*p* < 0.05, Figure 1D). Transmission electron microscope (TEM) images, Figure 2C–E, from lower to higher magnification, where labeled cells are indicated by red arrows, showed no discernible structural alteration. Furthermore, labeled cells were tested for the expression levels of chondrogenetic marker genes including Sox-9, aggrecan, and type II collagen with glyceraldehyde 3-phosphate dehydrogenase (GAPDH) as the internal control. Figure 3 demonstrates that phenotypical gene expressions remained unaltered even after 21 days of magnetic nanoparticle incorporation into the chondrocyte cells.

After cellular labeling, the cultures were verified as retaining their potential for effective chondrogenesis and osteogenesis by immunofluorescence with specific antibodies to related proteins such as α-smooth muscle actin (α-SMA), type II collagen, aggrecan, SRY-related high mobility group-box gene 9 (*Sox-9*), matrix metalloproteinase-13 (MMP-13), and osteonectin, as shown in Figure 3A–F, respectively. Nuclei were stained blue and cytoplasm stained green. The morphology of magnetically labeled chondrocytes appeared to keep the original circle shapes and showed no obvious effect on cell viability (Figure 1D), chondrogenesis, or osteogenesis as indicated by positive immunofluorescence staining (Figure 3). Hence, this result suggested chondrocytes labeled with magnetic nanoparticles might retain cell bioactivities.

### 2.2. Characterization of Biphasic Type II Collagen–Chitosan/Poly Lactic-co-Glycolic Acid (PLGA) Scaffold

**Figure 4:**
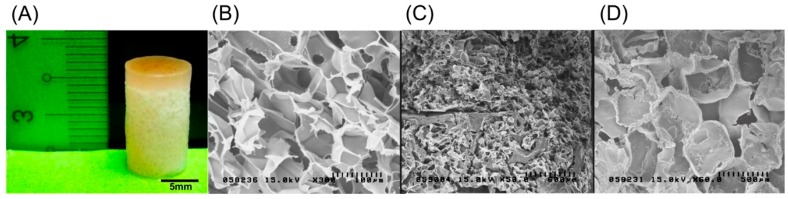
Photograph of an 8 mm × 12 mm biphasic type II collagen–chitosan/PLGA scaffold (**A**). Representative scanning electron microscopic images illustrating the open porous structure of the upper type II collagen–chitosan layer (**B**); interface region (**C**); and lower PLGA layer (**D**).

The biphasic scaffold, as shown in Figure 4A, had efficient integration between two layers to provide a compact structure as a whole with an independent environment upper type II collagen–chitosan layer with a pore size of 100 µm for cell attachment and chondrogenesis, and a lower PLGA layer with a pore size of 500 µm for osteogenesis, vascularization, and osteoconduction. SEM images of the three layers—the type II collagen–chitosan layer, the intermediate layer, and the PLGA layer—are shown in Figure 4B–D, respectively. The porous nature and interaction between the polymer components produced a contiguous interface between the chondrogenic and osteogenic polymers with good swelling properties.

### 2.3. Magnetic Nanoparticle-Labeled Chondrocyte Distribution under 1Ts Magnetic Field

**Figure 5:**
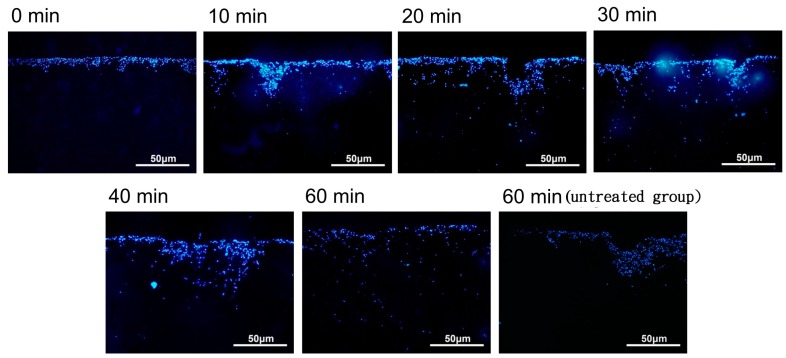
The distribution of magnetic nanoparticle-labeled cells cultivated into a type II collagen–chitosan/PLGA scaffold from 0 to 60 min under the influence of a magnetic field.

The cellular distribution of the nanoparticle-labeled chondrocytes placed in a magnetic field (1 Ts magnet) was observed after 0, 10, 20, 30, 40, and 60 min by fluorescence microscopy. When placed in a magnetic field (1 Ts magnet), the nanoparticle-labeled cells appeared to be distributed through the biphasic scaffold more evenly than in the untreated group (no magnetic field) (see Figure 5). However, there was variability across the time course, and further experiments are required to clarify whether the induced magnetic field assists in this regard.

### 2.4. In Vitro Chondrogenesis Study

**Figure 6:**
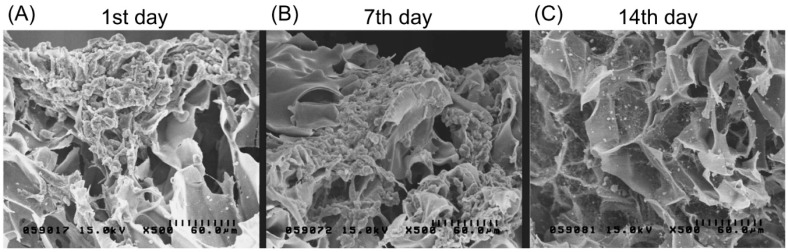
Scanning electron microscopic images showing chondrocytes (labeled with magnetic nanoparticles) grown on the type II collagen–chitosan/PLGA scaffold at 1, 7 and 14 days after seeding. Cell adhesion and spreading on the pores of the scaffold can be observed.

Magnetically labeled chondrocytes were grown on the biphasic scaffold up to 14 days. Figure 6A presents an SEM image showing no attachment of the cell culture on the scaffold after one day. Cells were only found gathered on the scaffold surface. After seven days, cells started to adhere and distribute, and proliferate deep into the biphasic scaffold surface. At 14 days, SEM showed extracellular matrix secretion as well as a greater accumulation on the surface with a better growth pattern and morphology.

**Figure 7:**
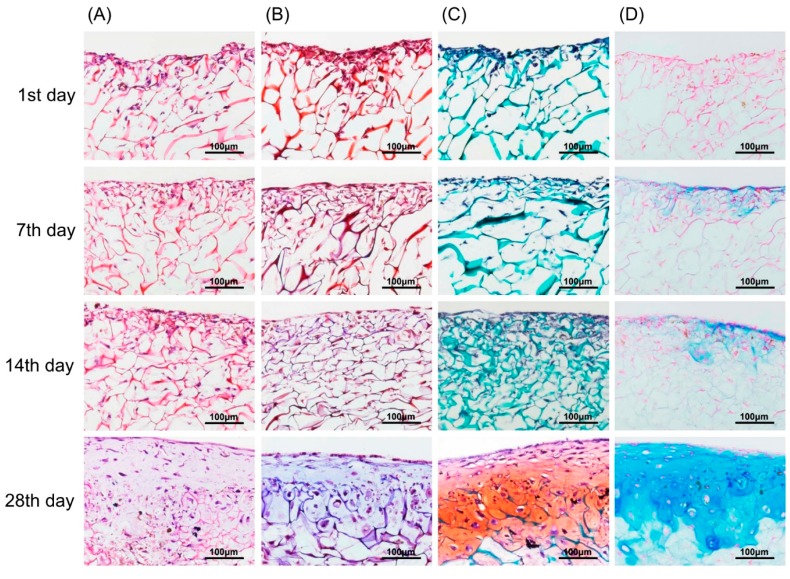
Chondrocytes (labeled with magnetic nanoparticles), morphology, and GAG distribution in the type II collagen–chitosan/PLGA scaffold from 1 to 28 days culture. H&E stain (**A**); Masson’s trichrome stain (**B**); Safranin O/Fast Green stain (**C**); and Alcian blue stain (**D**).

**Figure 8:**
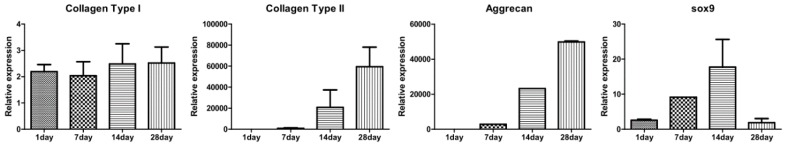
Gene expression in chondrocytes labeled with 250 µg/mL magnetic nanoparticles in the type II collagen–chitosan/PLGA scaffold cultured for 1, 7, 14 and 28 days. The expression of hyaline cartilage specific genes for collagen type II, aggrecan, and Sox-9, and osteogenic genes collagen type I (*n* = 3).

In vitro chondrogenic differentiation was visualized by various staining methods from Day 1 to Day 28. In Figure 7A, H&E stained images show a structural, chondrogenic differentiation pattern with maturation at 28 days. Masson’s trichrome stained images (Figure 7B) show a significant increase in total collagen content with progressive chondrogenesis. The Safranin O/Fast Green stain for cartilage (Figure 7C) shows a continuous uniform distribution of chondrocytes throughout the scaffold from 1 to 28 days covering maximum areas. Alcian blue stained glycosaminoglycans in cartilage (Figure 7D) showed increased color intensity and ECM formation from Day 1 to Day 28 with a remarkable lacunae-like structure formation. Real-time PCR for chondrogenesis (i.e., collagen type II, aggrecan, Sox-9) and osteogenesis (i.e., collagen type I) confirmed specific marker gene expression levels for these markers. As represented in Figure 8, the relative expression level of collagen type II significantly increased from Day 1 to Day 28. A remarkable upregulation of aggrecan is observed in Figure 8 at 14 and 28 days. Interestingly, upregulation of the *Sox-9* gene was found to be significant up to 14 days showing chondrogenesis, but decreased at 28 days, a sign of hypertrophy and osteogenesis. In contrast, no significant variation in expression level of collagen type I genes after 7-day cultivation was found. However, obvious upregulation occurred after 14-day cultivation. This result for collagen type I genes was correspondent to the behavior of the *Sox-9* gene in relation to the onset of osteogenesis.

## 3. Discussion

Osteochondral autologous transplantation is the treatment of choice for focal articular cartilage defects [2,5]. Mosaicplasty, the transplantation of autologous osteochondral plugs obtained from a non-weight bearing area of articular surface, is a well-established technique in clinical practice, especially for larger articular defect [16,17]. However, donor site mobility and the limited numbers of autografts obtainable from a patient necessitate investigations on possible substitutes for scarce osteochondral autografts.

Chitosan, a natural and biodegradable polysaccharide, has been widely used in biomedical applications, including cartilage regeneration [18]. Chitosan enhances formation of stable collagen–chitosan complexes due to abundant amino groups on its main chains [19]. Moreover, chitosan, with the same structural characteristics as glycosaminoglycans (GAGs), is thought to be an appealing GAG analog for cartilage repair [20]. Combined with type II collagen, chitosan can be an ideal biomimetic scaffold for cartilage tissue engineering [21]. PLGA is a synthetic polymer approved by the Food and Drug Administration (FDA) and has been widely utilized in the tissue engineering field, including as a slow-release biomaterial for drugs due to its biodegradability, biocompatibility, low immunogenicity, good mechanical strength, and general stability [22]. Success has been achieved with applications of PLGA in bone and cartilage tissue engineering in many in vitro and in vivo studies [9,10,23].

In order to mimic the mechanical characteristics and microenvironment of osteochondral plugs, a bi-layer scaffold composed of type II collagen–chitosan as the upper layer and PLGA as the lower was synthesized in this study. The layer of type II collagen–chitosan forms a chondrocyte-friendly environment and enhances seeded chondrocyte proliferation into cartilaginous tissue. The PLGA layer below is proposed to be a transition zone for chondrocyte migration, promoting bone and vascular ingrowth from recipient tissue in the osteochondral defects owing to its biocompatibility, high porosity, and hydrophilicity. The PLGA layer also offers mechanical stability for transplantation of the entire scaffold construct.

The application of magnetic nanoparticles has been widely utilized in cellular labeling and tracking, cell separation, drug delivery, magnetic resonance imaging, and magnetofection [12,13,24,25,26]. Magnetic nanoparticles are comprised of an iron oxide core coated with biological polymers facilitating uptake by targeted cells through endocytosis [27]. Several in vitro studies have reported that iron oxide-based magnetite nanoparticles do not affect the viability or proliferative capacity of chondrocytes [14,27]. However, long-term in vivo studies have not been performed extensively. In this study, we demonstrated the optimum concentration of magnetite nanoparticles (250 µg Fe_2_O_3_/mL) for labeling chondrocytes without altering cell morphology and viability after 21 days culture in vitro. Moreover, when magnetically labeled chondrocytes were seeded into the bi-layer scaffold under a one Tesla magnetic field for 60 min, chondrocytes showed a phenomenon that cell migrated and distributed evenly in the type II collagen–chitosan layer, facilitating the formation of cartilage tissue, but also migrated extensively deep into the PLGA layer. This biomimetic scaffold, resembling an osteochondral plug obtained by the mosaicplasty technique, is therefore proposed as an ideal substitute for repair of osteochondral defects without donor site mobility and limitations of graft source availability.

There is still controversy surrounding the possible inhibitory effect of magnetite nanoparticle labeling on chondrogenesis in mesenchymal stem cells [28,29]. However, in this study, we utilized chondrocytes instead of stem cells for cartilage engineering and demonstrated that these cells can be successfully labeled without phenotypic instability. The labeled chondrocytes can be successfully incorporated into cartilage tissue engineering systems using magnetic force. Differentiation of magnetically labeled mesenchymal stem cells and their incorporation into our biomimetic scaffold construct remains to be investigated. Moreover, although the in vitro results are highly encouraging, it also remains to be demonstrated whether the fate of this bi-layer scaffold engineered with magnetic particle-labeled chondrocytes can be monitored in vivo. In vivo experiments are already in progress and the results will be published separately.

## 4. Materials and Methods

### 4.1. Iron Oxide Magnetic Nanoparticles

Iron oxide magnetic nanoparticles TANBead^®^ USPIO-101(Amine group) were purchased from Taiwan Advanced Nanotech Inc. (Taipei, Taiwan). The concentration of magnetic nanoparticles was 1.4 × 10^16^ beads per mL at pH 3.8.

### 4.2. Isolation of Rabbit Chondrocytes

All animal procedures were conducted according to the “Guide for the Care and Use of Laboratory Animals” after approval by the Committee of Experimental Animal Sciences of Chang Gung Memorial Hospital (Approval No. 2011092802, date 22 October 2014). New Zealand White rabbits about 3 kg in weight were anesthetized with ketamine (Sigma, St. Louis, MO, USA) and xylazine (Bayer, Kyonggi-do, Korea) by intramuscular injection, and the knee cartilages were removed under aseptic condition. Cartilage pieces were finely minced, washed with PBS, and digested with 0.2% (*w*/*v*) collagenase (Sigma) in PBS for 5 h at 37 °C. The isolated chondrocytes were resuspended in DMEM/F12 (Thermo Fisher Scientific, Waltham, MA, USA) supplemented with 10% FBS (Thermo Fisher Scientific), 100 U/mL penicillin G (Thermo Fisher Scientific), and 100 μg/mL streptomycin (Thermo Fisher Scientific). The cells were plated at a density of 1 × 10^5^ cells/mL and placed in a 5% CO_2_ incubator at 37 °C with changes of medium on alternate days.

### 4.3. Evaluation of Rabbit Chondrocytes Labeled with Magnetic Iron Oxide Nanoparticles

Rabbit chondrocytes were cultured in 24-well culture plates (5 × 10^4^ cell /well, Thermo Fisher Scientific) in a 5% CO_2_ incubator at 37 °C for 24 h. The medium with various concentrations of magnetic nanoparticles (1000, 750, 500, 250, 100, 50, 25 and 0 µg Fe_2_O_3_/mL) were used for cell labeling. After cultivation for 48 h, cell morphologies were assessed by light microscopy, scanning electron microscopy, and transmission electron microscopy. The cells were washed twice with PBS and trypsinized. Finally, 1 mL of fresh medium was added into the tube for cell counting (total cell number). The tube was then placed in a magnetic field (1 Ts). Magnetically separated, labeled chondrocytes were counted again (labeled cell numbers). The extent of labeling was calculated as the ratio of the number of labeled cells to the total number of cells. The cell proliferation assay reagent WST-1 (Roche, Penzberg, Germany) was used to measure cell viability, cell cytotoxicity, and the relative proliferation activity of cells according to the manufacturer’s instructions. After several days of culture, growth medium was removed and cells were washed with PBS. Then, WST-1 (500 µL) at a volume ratio of 1:10 was added to the complete culture medium which had been prepared with DMEM without phenol red (Thermo Fisher Scientific). The WST-1 solutions were added to each well of the 24-well plate and incubated for 2 h at 37 °C in a 5% CO_2_ atmosphere. Dyed medium solution (150 µL) was placed into a 96-well plate (NUNC) for measuring absorbance. The absorbance at 450 nm was measured using an ELISA reader (Thermo Labsystems, Waltham, MA, USA). WST-1 solution was used as the blank reference. Three replicates were measured for each sample, and the mean and standard deviation were calculated. All experiments and analyses were performed for three independent experiments.

### 4.4. Protein and Gene Expression Profile for Chondrogenesis

For protein analyses, chondrocytes were cultivated in 24-well culture plates (2 × 10^4^ cell /well) in a 5% CO_2_ incubator at 37 °C for 24 h. The medium containing magnetic nanoparticles (250 µg Fe_2_O_3_/mL) was used for cell labeling. After 72 h cultivation, the cells were methanol-fixed for 15 min and then prepared for immunofluorescence microscopy via staining with specific antibodies (α-SMA, collagen type II, aggrecan, Sox-9, MMP13 and osteonectin). In the case of gene expression analyses, chondrocytes were cultivated in 24-well culture plates (2 × 10^4^ cell /well) in a 5% CO_2_ incubator at 37 °C for 24 h and then labeled by adding medium containing magnetic nanoparticles (250 µg Fe_2_O_3_/mL). After 24 h cultivation, the medium was changed again to the original medium for cultivation. The cells were harvested at predetermined intervals (days 1, 3, 7, 14, and 28) for gene expression analysis by quantitative real-time PCR. All experiments and analyses were performed for three independent experiments.

### 4.5. Preparation and Characterization of the Biphasic Type II Collagen–Chitosan/PLGA Scaffolds

APLGA solution (5% *w*/*v*) was prepared by dissolving PLGA (Lactide/Glycolide = 85/15, MV = 230,000, PURAC) in 1,4-dioxane. NaCl (7 g, grain size: 500 µm) was added and mixed. The well-mixed solution was injected into molds (10 mm × 8 mm) and then lyophilized. After lyophilization, NaCl was washed out using distilled water to create porous structures. Chitosan solution (2% *w*/*v*) and type II collagen solution (2% *w*/*v*) were mixed to form a of chitosan–collagen type II solution (1% *w*/*v*). The pre-prepared PLGA scaffolds were placed into molds and the chitosan–collagen type II solution was injected into the same mold. After lyophilization, fabrication of the biphasic, chitosan–collagen type II/PLGA scaffolds was complete. All experiments and analyses were performed for three independent experiments.

### 4.6. Magnetic Field Treatment for Magnetic Nanoparticle-Labeled Chondrocytes on Biphasic Type II Collagen–Chitosan/PLGA Scaffolds

Chondrocytes were cultivated in a 24-well culture plate (1 × 10^6^ cells /well) in a 5% CO_2_ incubator at 37 °C for 24 h. The medium was changed to one incorporating magnetic nanoparticles (250 µg Fe_2_O_3_/mL) for cell labeling. After cultivation for 24 h, the cells were washed twice with PBS and trypsinized. Finally, 1 mL of fresh medium was added into the tube and placed into a magnetic field at 1 T. The separated chondrocytes were counted to prepare a concentration of 1 × 10^7^ cell/mL for further use. The biphasic scaffolds were sterilized using H_2_O_2_ plasma. Labeled cells were cultivated on the surface of the scaffolds that were then placed into a magnetic field for 0, 10, 20, 30, 40, or 60 min. After magnetic field treatment, the scaffolds were taken out, fixed with 10% formaldehyde, and stained with 2 mL Hoechst 33,258 to observe the cell distribution and to evaluate the optimal magnetic field treatment duration for uniform distribution of labeled cells. All experiments and analyses were performed for three independent experiments.

### 4.7. Cell Morphology Study after Magnetic Field Treatment of Labeled Chondrocytes on Type II Collagen–Chitosan/PLGA Scaffolds

Chondrocytes were cultured in 24-well culture plates (1 × 10^6^ cells /75T flask) in a 5% CO_2_ incubator at 37 °C for 24 h. The medium containing magnetic nanoparticles (250 µg Fe_2_O_3_/mL) was used for cell labeling. After 24 h cultivation, the cells were trypsinized and cultivated on the surface of biphasic scaffolds. After magnetic field treatment, the cells were harvested at the predetermined interval (Days 1, 7, 14, and 28) for the cell morphology, distribution, and expression of particular genes.

### 4.8. Histology

Specimens were harvested and fixed in 4% paraformaldehyde solution (Sigma) at 4 °C overnight and then stored in 70% alcohol at 4 °C. The specimens were embedded in paraffin, sectioned to a 5 μm thickness, and processed by hematoxylin and eosin staining to distinguish osseous tissue phenotypes. Calcium deposition of the tissue constructs was visualized after H&E stain, Masson’s trichrome stain, safranin O stain, and Alcian blue stain. The sections were viewed under a light microscope (Olympus, Tokyo, Japan) for histological evaluation. All experiments and analyses were performed for three independent experiments.

### 4.9. Real Time PCR

Cell-laden scaffolds with chondrogenic medium were analyzed by real-time PCR using the SyBr green system to investigate mRNA expression changes in real time of collagen type I, collagen type II, aggrecan and Sox-9. The specified primers were as follows: collagen type I-F: GATGGTCAGCCTGGACACA; R: CGAAGGCCAGCAGGTCCAA and collagen type II-F: CTGAGGGCTCACGCAAGAA; R: GCAGCACGGTATAGGTGAA and aggrecan-F: CAGGAGGCAGCCAGTGAGT; R: GACGTCCAGCACCGGCTT and Sox-9-F: TTCATGAAGATGACCGACGA; R: CACACCATGAAGGCGTTCAT. The total mRNA extraction was performed following the reported protocols. The reaction mixture was kept at 95 °C for 10 min, followed by real-time PCR for 40 cycles. Each cycle included denaturation at 95 °C for 20 s, followed by annealing and extension at 61 °C for 1 min. Glyceraldehyde phosphate dehydrogenase (GAPDH-F: GAGCTGAACGGGAAACTCAC; R: GGTCTGGGATGGAAACTGTG)) was used as the internal control. The comparative CT method was used for quantitative gene expression studies. All experiments and analyses were performed for three independent experiments.

### 4.10. Statistical Analysis

All experiments and analyses were performed for three independent experiments. The gene expression data were presented as the means ± standard deviation (SD) of three experiments (*n* = 3) for each test. The control and experimental groups were compared using Student’s *t*-test.

## 5. Conclusions

In conclusion, this study demonstrates an alternative and efficient method to guide the distribution and proliferation of magnetically labeled chondrocytes within a bi-layer, biomimetic scaffold. This construct has potential for the development of tissue engineering-based strategies for cartilage repair and regeneration. These novel cell/biomaterial constructs are anticipated to improve the repair of articular cartilage defects and should be readily transferable from the laboratory bench top to a clinical setting in the future.
